# The impact of major public health emergencies on Trust in Government: From SARS to COVID-19

**DOI:** 10.3389/fpsyg.2022.1030125

**Published:** 2022-11-16

**Authors:** Kun Zhai, Xuemei Yuan, Guoqing Zhao

**Affiliations:** ^1^Business School, Institute of Finance, University of Jinan, Jinan, China; ^2^Shandong Key Laboratory of Blockchain Finance, Shandong University of Finance and Economics, Jinan, China

**Keywords:** public health emergencies, trust in government, epidemic experience, age group, long-term effect

## Abstract

Major public health emergencies always test the credibility of the government. The success of governments’ strategies relies on trust in government and broad acceptance of response measures. The profound experience of the epidemic often has a long-term impact on people’s cognition. We construct a difference-in-difference estimator by combining the variations of epidemic effects across cohorts and regions, and intend to evaluate the long-term effect of individuals’ early SARS experience on trust in government during the COVID-19 pandemic. We also use the instrumental variable method to overcome the endogenous problem caused by two-way causality. The results show that the impact of COVID-19 has significantly reduced trust in government of the groups who had not been exposed to the SARS epidemic (including groups who were in early childhood and the unborn during the SARS outbreak). While it has a positive impact on trust in government of people experienced SARS in adolescence, and only a little negative impact on trust in government of people experienced SARS in adulthood. We also find that the impact of COVID-19 mainly reduced the trust in government among groups socially vulnerable or without SARS experience (e.g., low income, low social status etc.). The results suggest that: (a) the trust created by governments’ successful anti-epidemic measures is long-lasting; (b) governments should pay more attention to their trust among socially vulnerable groups.

## Introduction

Since 2000, many infectious diseases have broken out worldwide, such as severe acute respiratory syndrome (SARS), H1N1, and Ebola. These diseases not only cause death but also extensive and long-term social and economic chaos (Ye and Lyu, 2020). COVID-19, one such infectious disease, was first reported in Wuhan, China, in December 2019 (Shaw et al., 2020). It spread in China from December 2019 to early 2020 and then quickly spread to other countries. The Chinese government has implemented unprecedented quarantine measures, leaving a large number of people in isolation and affecting many aspects of people’s daily lives (Qiu et al., 2020). There are significant differences between countries’ measures of stopping the spread of COVID-19. Some countries took strict epidemic control policies supported and implemented by the public, while other countries tended to rely more on suggestions with little effect. The difference of trust in government may reveal the social dilemma behind the spread of COVID-19.

Trust is a key factor for successfully implementing a government’s policies. Citizens must believe that the recommendation from the authorities are correct and maximize the collective interests. So most people will follow these suggestions (Harring et al., 2021). Local governments can play a key role in protecting citizens in the event of major disasters (Col, 2007). The foundation for a government to play this important role is the public’s trust (Mansoor, 2021). The unexpected outbreak of COVID-19 prompted many governments to implement preventive measures to curb the spread of COVID-19 (Fetzer et al., 2020). During the COVID-19 crisis, if a country’s people had low trust in government, the country’s resilience declined (Kimhi et al., 2020). There is also evidence that trust in local governments can reduce the infection rate of COVID-19 (Ye and Lyu, 2020).

In 1964, 75% of Americans believed that their government had done the right thing in most cases. By 1995, only 15% thought so. In 1985, 8.5% of New Zealanders were full of confidence in government. By 1998, the proportion had dropped to 2.5% (Barnes and Gill, 2000). Most research cites transnational politics as the reason for the decline; so trust or a lack of trust is mainly explained by population, society and political trend (Bovens and Wille, 2008). However, major public health emergencies provide a new explanation for the change in residents’ trust in government. At present, the relevant research mainly focuses on the impact of a major public health emergency on people’s trust in government, and few studies have examined the impact of multiple major public health emergencies on people’s trust in government. Considering SARS and COVID-19, this paper discusses the impact of COVID-19 on trust in government of people with and without prior exposure to an epidemic. This paper attempts to explain the inconsistent conclusions on the impact on trust in government during an epidemic and successful epidemic control in China.

At present, the impact of major public health emergencies on residents’ trust in government is inconsistent across countries. When people are affected by disasters, it is generally believed that their trust in government will decline (Hommerich, 2012; Kimhi et al., 2020; Sibley et al., 2020). From 2014 to 2015, Ebola virus spreaded rapidly in Liberia. The worsening of the epidemic was not only due to the poor response of government but also due to the citizens’ lack of trust in government. They were suspicious of the virus’s source and spread, and even refused to wash their hands regularly or do other simple preventive measures, which exacerbated the epidemic. The suffering experience during the crisis destroyed people’s trust in government, taking the epidemic from “a health crisis to a governance crisis” (Moxham-Hall and Strang, 2020). Ma and Christensen (2019) found that citizens’ trust in the central government was significantly negatively correlated with perceived emergencies (e.g., public events, public health threats), while trust in the local government was slightly positively correlated with perceived emergencies. Paek et al. (2008), through a telephone survey, found that more than half of the respondents believed that the government could cope with the influenza pandemic and expressed strong support for many actions proposed by the government during the influenza pandemic. During the outbreak and worsening of COVID-19, people in 58 countries expressed complaints about governments’ responses to the virus for they thought the governments’ responses were insufficient (Hale et al., 2020), thus reducing the public’s trust in government (Chen and You, 2021; Adamy and Rani, 2022). Aksoy et al. (2020) proposed that under governments with less capability against the epidemic, epidemic exposure in an individual’s “impressionable years” (ages 18 to 25) would have a persistent negative effect on confidence in political institutions and leaders. Public confidence in government rises or falls with the perception of the country’s ability to respond to major disasters. A major crisis can be a crucial moment for consolidating governments’ political status quo. When a government announces measures in the best interests of the public, the trust among citizens changes positively (Yousaf et al., 2016; Galle et al., 2020). A government’s decisions in emergency situations, such as stay-at-home orders, free medical facilities, and financial assistance, will increase the public’s trust in government, especially when the government gives a strong response. The Ebola epidemic in West Africa enhanced trust in government (Fluckiger et al., 2019). The COVID-19 crisis led to a higher level of institutional trust in Sweden (Esaiasson et al., 2020) and more trust in the central and local governments of South Korea (Kye and Hwang, 2020). Comparing the people who were isolated during COVID-19 with those without isolation, it is found that the people who were isolated had higher trust in politicians (Sibley et al., 2020) and government (Bol et al., 2020; Groeniger et al., 2021). These results indicate that in major public health crises, people appreciate the government’s resolute, early and rapid intervention.

The efficiency of policies in reducing liquidity increases significantly with trust. This trust effect is nonlinear and increases with policy strength (Bargain and Aminjonov, 2020). Trust is the key to the public’s compliance with policies aimed at controlling epidemics (e.g., physical isolation, vaccination). People who trust the government are more likely to comply with the rules (Blair et al., 2022; Rauf et al., 2022; Shanka and Menebo, 2022), so it is the basis of social interaction (Uslaner, 2002; Turgay, 2022) and decision-making (Yamagishi and Kiyonari, 2000). During the COVID-19 epidemic, the lack of trust may lead to nonvaccinated people and noncompliance with preventive measures, which may overwhelm the medical system (Stefaniak et al., 2022).

The public’s trust in government in the early stage of an epidemic has an important impact on whether the crisis can be successfully managed in later stages. Although this is an important issue, there are few relevant studies. Freimuth et al. (2014) found that the public had low trust in government in the early stage of H1N1, and this lack of trust predicted the lag in vaccine acceptance. Eichengreen et al. (2021) showed that epidemic exposure (at ages 18–25) had a negative impact on the confidence of current scientists.

This paper argues that residents’ past experiences have forged their current cognition, forms stable government trust status, and thus affects the efficiency of society. The duration of SARS in China was from the end of 2002 to the middle of 2003, lasting approximately 7 months. By 2020, Chinese residents aged 24 and above were considered exposed to the SARS epidemic with a clear understanding of the government’s prevention and control capabilities. From the perspective of the two major epidemics in China, combining real-time big data and data from the China Family Panel Studies (CFPS) conducted in 2020, this paper divides the age of the respondents into the group that has not been exposed to SARS and the group that has been exposed to SARS and studies the impact of COVID-19 on the trust in government on both groups. We find that there is a significant difference between exposure to SARS in adolescence (7–17 years old) and exposure to SARS in adulthood (aged 18 and above). Compared with the literature, the contribution of our paper can be clarified in three aspects. First, the vast majority of the literature studies the impact of major epidemics on the public’s trust in government, only considering the current government’s governance and responses, but few studies are conducted from the perspective of individual epidemic experience. Our study may be the first to evaluate the long-term effect of SARS epidemic experience on residents’ trust in government during the COVID-19 epidemic, which is of great importance to government’s management and epidemic prevention and control. Second, we construct a difference-in-difference (DID) estimator by combining the variations of epidemic effects across cohorts and regions, using the instrumental variable method to overcome the impact of endogenous problems caused by two-way causality on the estimation results. Third, we consider the impact of the COVID-19 epidemic on trust in government of different groups with or without SARS experience, and try to reveal the mechanism of the epidemic on trust in government. This paper properly explains the different policy implementation efficiencies of governments under the impact of COVID-19.

The rest of this paper is arranged as follows: the second section is materials and methods. The third section shows the main regression results, specification tests and the heterogeneity analysis. The fourth section is the robustness test. Section 5 further discusses the mechanisms.

## Materials and methods

### Materials

The paper applies an original dataset combining real-time big data, official data, and CFPS data. CFPS is implemented by the China Social Sciences Research Center of Peking University. The CFPS focuses on the economic and noneconomic welfare of Chinese residents, as well as many research topics, including economic activities, educational achievements, family relations and family dynamics, population migration, and health. It is a national, large-scale, multidisciplinary social follow-up survey project. In 2010, CFPS officially implemented a baseline survey in 25 provinces (municipalities directly under the Central Government and autonomous regions) across the country. The population of these 25 provinces (municipalities directly under the Central Government and autonomous regions) accounts for about 95% of the Chinese population (excluding Hong Kong, Macao and Taiwan), so the CFPS can be regarded as a representative sample of China. Since 2010, CFPS has conducted a full sample tracking survey every 2 years (CFPS 2012, 2014, 2016, 2018, 2020). In the sampling method, CFPS adopts implicit stratified, multi-stage, multi-level, proportional to population size probability sampling (PPS), and administrative divisions and socio-economic levels are the main stratified variables. In 2020, CFPS covered 22 provinces (excluding Taiwan province only), 4 municipalities directly under the Central Government and 5 autonomous regions, with a sample size of 28,590 individuals. At present, CFPS has only published the 2020 personal-level database.

### Main variables

#### Explained variable

Trust is a complex construct with multiple dimensions, making it difficult to define and operationalize (Simpson, 2007; Sanchez et al., 2012). Trust in government means that the government is believed to be correct and fair in performing its duties, public communication and other behaviors (Brewer and Sigelman, 2002), and the implementation of such behaviors is carried out by government officials. Wang (2017) believed that in China, government officials represent the government to a certain extent, and their behavior, attitude and ability affect citizens’ comprehensive judgment of the government in the process of interaction with citizens. To a large extent, the public’s trust in government can be expressed through the trust in government officials.

There is no direct investigation on trust in government in CFPS. This paper refers to Shen and Zhou (2017), using trust in government officials as the proxy variable of trust in government. The variable “Trust in Government” is from CFPS. “Trust in Government” is obtained by investigating the question “How much do you trust local government officials?” Responses ranged from “totally distrust” to “totally trust” on a scale with 11 choices (0 points means do not trust them at all, 10 points means trust them a great deal).

#### The Key explanatory variables

##### Cumulative number of confirmed infections

The 2020 CFPS survey was conducted from July to December 2020. This paper mainly utilizes the cumulative number of confirmed infections (CNCI; including cured and deceased individuals) on July 1, 2020, in each province for the analysis. The data comes from the real-time big data released by the Chinese government.

##### Age group (Agegr)

This paper studies whether the influence of COVID-19 on trust in government is different with individuals exposed to the SARS epidemic at different ages. Accordingly, we classify the age of respondents from 2002 to 2003 and then divided them into different age stages according to the growth stage of human beings. Referring to Zhan (2002), the whole childhood of human beings is divided into infancy (less than 7 years old), childhood and adolescence (between 7 and 17 years old; Agegr_2_). The setting of adulthood is 18 years old and above (Agegr_3_). In this part, infants and unborn children are classified as the control group. See Table 1.

**Table 1:** Division of birth age stage of respondents.

Age at the time of investigation	Year of birth	Age in 2002–2003	Life cycle in 2002–2003	Abbreviation
<24 years old	(1996—	<7 years old	childhood	Agegr_1_
[24,35)	(1985—1996]	[7,18)	adolescence	Agegr_2_
> = 35 years old	—1985]	> = 18 years old	adulthood	Agegr_3_

##### Descriptive statistics

The main driving factor of trust in government is individuals’ perception of government performance and the social and economic environment in which they live (García and Gaytán, 2013). It is generally believed that political corruption reduces trust in government (Catterberg, 2006; Wang, 2010) and political trust (Anderson and Tverdova, 2003; Chang and Chu, 2006; Beesley and Hawkins, 2022). It seems that the integrity of government is a relatively important factor affecting trust in government, so we add the clean government variable as a control variable. It is obtained from the question “How serious do you think the problem of government corruption is in China?” (0 means not serious at all, and 10 means the most serious).

For other micro variables, citizens’ satisfaction with public services (such as education, medical care, and public security) is important in their evaluation of government performance, and personal satisfaction with income, happiness, and work is also very important. In terms of macro variables, the economic situation of a country seems to be crucial. If a country’s economy grows rapidly, citizens are more likely to be satisfied with the government’s performance (Wang, 2010). Generally, governments’ performance and trust in government are positively correlated. In addition, Chen and You (2021) considered that an increase in the pollution level would reduce citizens’ trust in government. We consider these factors in the control variables.

Control variables mainly include demographic variables and variables at the provincial level. Demographic variables are collected in the CFPS, and variables at the provincial level are from the China Statistical Yearbook of 2020. Demographic variables include marriage, health, happiness, education, sex, medical insurance, working income, low income, high income and clean government. Among these variables, the health variable is obtained according to the question “What do you think of your health?” There are 5 levels, where 1 means very unhealthy and 5 means very healthy. The education variable is assigned according to the number of years of education that respondents have completed the highest degree. Specifically, when a respondent has never been to school or is illiterate (semi illiterate), the education level is assigned as 0; when the highest education of respondents is primary school, education is assigned as 6; when the highest education of respondents is junior high school, education is assigned as 12; when the highest education of respondents is high school, technical secondary school, technical school or vocational high school, education is assigned as 15; when the highest degree of interviewees is college or university undergraduate, education is assigned as 19; when the highest degree of respondents has a master’s degree, education is assigned as 22; when the highest degree of respondents has a doctor’s degree, education is assigned as 25. The happiness variable is obtained according to the question “How happy are you?.” There are 11 levels, where 0 means the lowest happiness, and 10 means the highest happiness. The employment variable is obtained from the question “How serious do you think the employment problem is in China?” (0 means nothing serious, and 10 means the most serious). The clean government variable is obtained according to the question “How serious do you think the problem of government corruption is in China” (0 means nothing serious, and 10 means the most serious). The life changes variable is obtained according to the question “Do you agree that in today’s society, people still have a great chance to improve their living standards?.” There are 5 levels, where 1 means disagree very much and 5 means agree very much. The social values variable is measured on the same scale and obtained in response to the question “Do you agree that hard work can be rewarded nowdays?”

Provincial variables included CPI, *per capita* GDP, and living conditions. The variable of living conditions is measured by the amount of municipal solid waste cleared. Descriptive statistics are given in Table 2.

**Table 2:** Descriptive statistics of the data.

Variable	Obs	Mean	Std.Dev.	Min	Median	Max
Trust in Government (TIG)	8,414	5.6963	2.3282	0.0000	5.0000	10.0000
CNCI(ten thousands)	8,414	0.1513	0.7543	0.0001	0.0595	6.8135
Provincial infection rate (IR)	8,414	0.0025	0.0127	0.0000	0.0008	0.1150
Provincial death toll (DT; Divide by 100)	8,414	0.4032	3.0740	0.0000	0.0300	27.6100
Age	8,414	32.4006	7.4989	16.0000	32.0000	85.0000
Sex (male =1)	8,414	0.5083	0.5000	0.0000	1.0000	1.0000
Education	8,414	13.9994	4.6494	0.0000	15.0000	25.0000
Marriage (Yes = 1)	8,414	0.7373	0.4401	0.0000	1.0000	1.0000
Health	8,414	3.4205	1.0097	1.0000	3.0000	5.0000
Medical insurance (Yes = 1)	8,414	3.4205	1.0097	1.0000	3.0000	5.0000
Working income (10,000 yuan)	8,414	3.2691	4.5167	0.0000	2.0000	70.0000
Low-income (Yes = 1)	8,414	0.2242	0.4170	0.0000	0.0000	1.0000
High-income (Yes = 1)	8,414	0.1745	0.3795	0.0000	0.0000	1.0000
Happiness	8,414	7.5257	1.8458	0.0000	8.0000	10.0000
Clean government	8,414	6.2558	2.5576	0.0000	6.0000	10.0000
Social values	8,414	5.0389	1.5395	1.0000	5.0000	8.0000
Life chances	8,414	3.8347	0.7036	1.0000	4.0000	5.0000
CPI	8,414	102.8268	0.3811	101.9000	102.9000	103.7000
*Per capita* GDP (10,000 yuan)	8,414	6.5240	3.1294	3.2995	5.6388	16.4220
Living conditions	8,414	10.8347	8.7129	0.6470	8.0220	33.4730

### Methods

A sudden major public health event has an exogenous impact on individuals, so it is a random natural experiment. Inspired by Chen and Zhou (2007) and Archibong and Annan (2017), we combined the variations of epidemic effects across cohorts and regions to construct a DID estimator. The first difference in this paper is the difference in the severity of COVID-19 epidemic among provinces; the second difference is using the age specific cohort to construct the difference of experiencing the SARS epidemic. In the third part of this paper, the hypothesis test of DID is carried out.

Considering that the explained variable “trust in government” is an ordered variable, in order to evaluate the impact of sudden major epidemics on trust in government, we adopt an ordered probit (Oprobit) model with a standard error of robust clustering at the provincial level and add an age-fixed effect to the regression equation. Specifically, the model is set as follows:


(1) $$\begin{array}{l} Y_{iap}^{*}=\alpha +\beta_{1}CNCI_{p}+\overset{3}{\underset{a=2}{\sum }}\beta_{a}CNCN_{p}^{}*Agegr_{ia}+\overset{3}{\underset{a=2}{\sum }}\gamma_{a}Agegr_{ia}\\ +\lambda_{a}+X_{iap}\varphi +Z_{ip}\Psi +\varepsilon_{iap}=\theta_{ip}+\varepsilon_{iap} \end{array}$$

$$\begin{array}{c} P\left(Y_{iap}=j|Controls\right)=P\left(cut_{j}<Y_{iap}^{*}\leq cut_{j+1}|Controls\right),\\ j=0,1,\ldots ,10. \end{array}$$

where the explained variable *Y_iap_* indicates the trust in government of individual *i* in province *p* and age group *a*. $Y_{iap}^{*}$ is the potential trust in government (latent variable) of individual *i* in province *p* and age group *a*. *Controls* represents the set of explanatory variables. *CNCI_p_* indicates the cumulative number of confirmed infections in province *p* as of July 1, 2020. *Agegr_a_* represents dummy variables for different age groups (see Table 1 for details). *λ_a_* represents the age-fixed effect, which controls the difference in individual trust in government caused by different ages. *X_iap_* represents a series of individual-level control variables. *Z_ip_* represents a series of provincial-level control variables. *ε_iap_* represents the random error term, and the clustering robust standard error at the provincial level is adopted. The threshold *cut_1_-cut_10_* are parameters to be estimated, where *cut*_0_ = −∞, *cut*_11_ = +∞_._

In this paper, the regression coefficients *β*_2_ and *β*_3_, show the long-term effect of the COVID-19 epidemic on trust in government among individuals who were not exposed to SARS and those who were exposed to SARS at different ages in different provinces.

## Results

### Data analysis

#### The changing trend of trust in government

We sorted out Chinese government’s trust data from the World Values Survey (WVS). To date, the WVS has conducted seven surveys. Among them, surveys 2, 4 and 7 involved Chinese government’s trust survey. We collect responses to two questions: trust in the central government and trust in political parties on a 4-point scale from 1 (completely mistrust) to 4 (completely trust).

**Figure 1:**
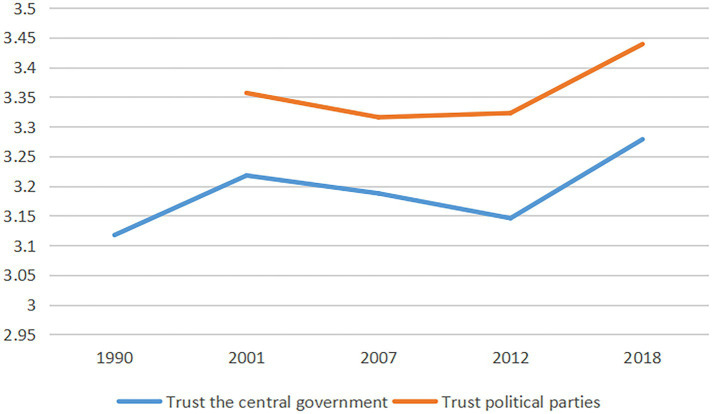
Trust in the Chinese government from 1990 to 2018.

As shown in Figure 1, from 1990 to 2018, residents’ trust in the central government and political parties shows a highly similar trend (trust in political parties was not investigated in 1990). Although some studies show that trust in government has dropped sharply in developed countries, trust in government is still high in China. However, for nearly 10 years from 2001 to 2012, residents’ trust in the Chinese government declined. This is consistent with the research of Zhao and Hu (2017), who found that Chinese citizens’ trust in government is much lower than in previous studies.

SARS occurred in China from 2002 to 2003. The experience of a major public health event may affect residents’ trust in government. We cannot infer that the occurrence of SARS is related to the decline of trust in government from the data alone. Considering the COVID-19 epidemic that occurred at the end of 2019, this paper obtains trust in government data from China Family Panel Studies (CFPS) in 2018 and 2020 (where trust in local government officials is scored from 0 (do not trust at all) to 10 (trust them a great deal). Trust in government in 2018 and 2020 was 5.0018 and 5.8509, respectively. When people experience major public health events again, trust in government does not seem to be reduced correspondingly.

#### Distribution of SARS and COVID-19

SARS first appeared in Guangdong, China, in 2002, then spread to Southeast Asia and the rest of the world. It was not until the middle of 2003 that the epidemic was gradually eliminated. There were 8,422 cases in the world, involving 32 countries and regions. A total of 5,327 SARS cases and 349 deaths were reported in mainland of China, although Heilongjiang, Hainan, Guizhou, Yunnan, Qinghai, Tibet and Xinjiang provinces reported no SARS cases.

**Figure 2:**
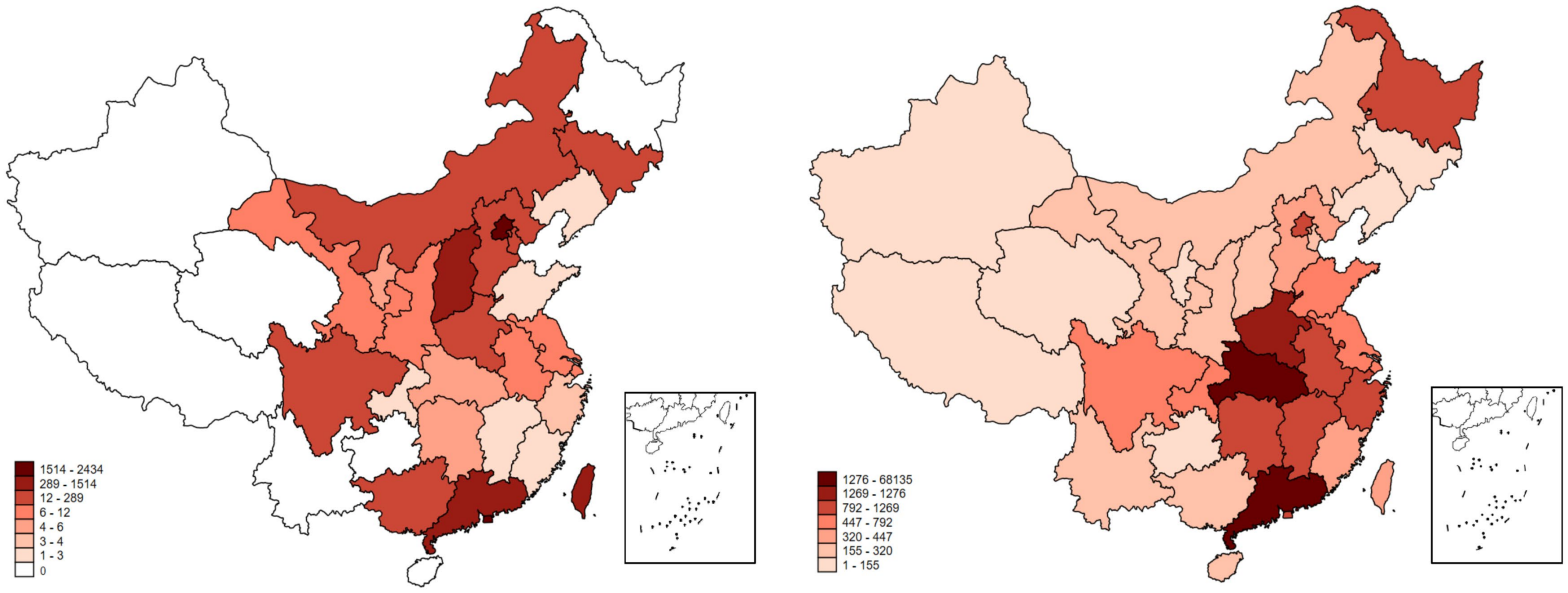
Distribution of the epidemic in China.

At the end of 2019, COVID-19 was first reported in Wuhan, Hubei Province, China. By midnight on July 1, 2022, a total of 83,536 confirmed cases and 4,634 deaths had been reported in 31 provinces (autonomous regions and municipalities directly under the central government). We collate the number of SARS infections of each province by May 28, 2003, and the number of confirmed COVID-19 cases of each province by July 1, 2020. Their distribution is shown in Figure 2 (the left figure shows the distribution of SARS; the right figure shows the distribution of COVID-19).

**Figure 3:**
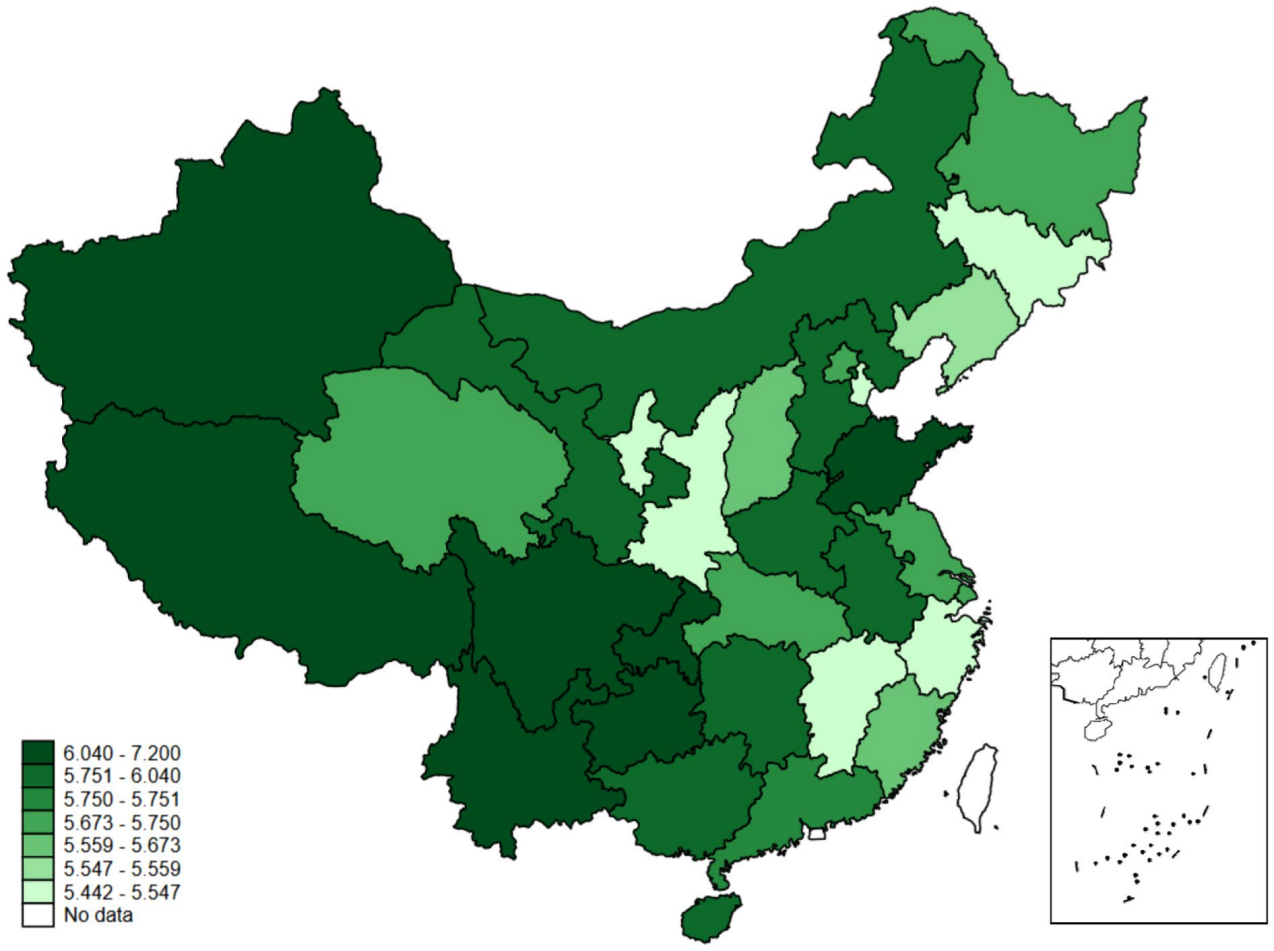
Distribution of residents’ trust in the Chinese government.

According to the CFPS survey data in 2020, we calculate the average value of residents’ trust in government in each province. This distribution is shown in Figure 3, which demonstrates that trust in government is high in Western China and Shandong Province. Hubei Province does not have low trust in government, even though it was the first to report COVID-19, and the number of infections is extremely high.

#### Distribution of trust in government among different age groups

**Figure 4:**
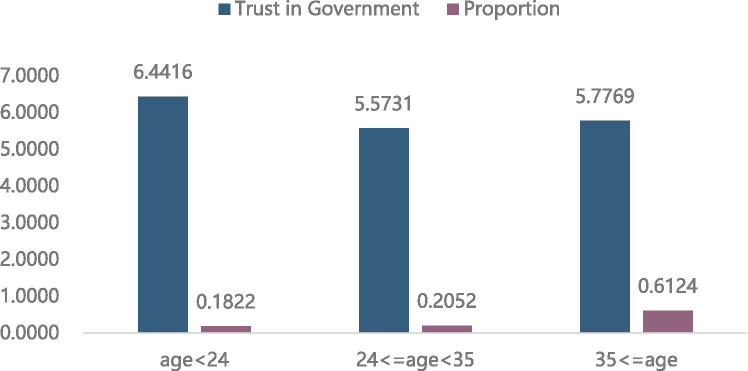
Distribution of trust in government in different age groups.

As shown in Figure 4, the group younger than 24 years has the highest trust in government (6.4416 points), accounting for approximately 18.22% of the total sample. The group aged 24 to 35 has the lowest trust in government (5.5731 points), which accounted for 20.52% of the total sample. Trust in government of individuals over age 35 is between the above two groups, with the largest sample size accounting for 61.26%.

#### Main empirical results

The estimated results are shown in Table 3. The first row of the regression results shows the impact of the COVID-19 epidemic on trust in government among people who were not exposed SARS. From the empirical results, we can see that the impact of COVID-19 has reduced trust in government among people who were not exposed to SARS. The main concern of this paper is that when a major public health emergency occurs again, groups that were not exposed to SARS and those were exposed SARS will have different levels of trust in government. Rows 2–3 of the regression results in Table 3 show the impact of the COVID-19 epidemic on trust in government among people who were exposed to SARS at different ages.

**Table 3:** Main regression results.

	(1)	(2)	(3)	(4)	(5)	(6)
	TIG	TIG	TIG	TIG	TIG	TIG
CNCI	−0.0548^***^	−0.0596^***^	−0.0597^***^	−0.0524^***^	−0.0582^***^	−0.0588^***^
	(0.0053)	(0.0074)	(0.0081)	(0.0049)	(0.0080)	(0.0082)
CNCI^*^Agegr_2_	0.0711^***^	0.0707^***^	0.0713^***^	0.0691^***^	0.0682^***^	0.0689^***^
	(0.0073)	(0.0061)	(0.0073)	(0.0068)	(0.0058)	(0.0067)
CNCI^*^Agegr_3_	0.0439^***^	0.0496^***^	0.0443^***^	0.0423^***^	0.0466^***^	0.0424^***^
	(0.0066)	(0.0062)	(0.0067)	(0.0069)	(0.0067)	(0.0069)
Agegr_2_	0.0395	−0.0147	0.0394	0.1510	0.0131	0.1531
	(0.1269)	(0.0332)	(0.1278)	(0.1272)	(0.0343)	(0.1288)
Agegr_3_	−0.3299^***^	0.0357	−0.3488^***^	−0.2253^*^	0.0700^*^	−0.2487^**^
	(0.1198)	(0.0399)	(0.1223)	(0.1198)	(0.0413)	(0.1223)
Marriage	−0.1468^***^	−0.1619^***^	−0.1468^***^	−0.1855^***^	−0.1945^***^	−0.1858^***^
	(0.0381)	(0.0350)	(0.0387)	(0.0330)	(0.0326)	(0.0339)
Health	0.0462^***^	0.0465^***^	0.0460^***^	0.0494^***^	0.0493^***^	0.0494^***^
	(0.0079)	(0.0080)	(0.0079)	(0.0074)	(0.0079)	(0.0074)
Happiness	0.0988^***^	0.0982^***^	0.0985^***^	0.0998^***^	0.0988^***^	0.0996^***^
	(0.0058)	(0.0055)	(0.0057)	(0.0060)	(0.0057)	(0.0059)
Education	−0.0002	−0.0009	−0.0004	0.0005	−0.0002	0.0003
	(0.0084)	(0.0076)	(0.0082)	(0.0096)	(0.0085)	(0.0092)
Sex	0.0064	0.0075	0.0077	−0.0056	−0.0036	−0.0045
	(0.0191)	(0.0178)	(0.0189)	(0.0195)	(0.0183)	(0.0195)
Medical insurance	0.1121^***^	0.1121^***^	0.1151^***^	0.1255^***^	0.1249^***^	0.1290^***^
	(0.0321)	(0.0316)	(0.0317)	(0.0298)	(0.0294)	(0.0299)
Working income	0.0055^*^	0.0050^*^	0.0050	0.0049	0.0045	0.0044
	(0.0031)	(0.0030)	(0.0032)	(0.0033)	(0.0032)	(0.0034)
Low-income	−0.1057^***^	−0.1033^***^	−0.1066^***^	−0.0977^***^	−0.0957^***^	−0.0990^***^
	(0.0317)	(0.0309)	(0.0316)	(0.0297)	(0.0295)	(0.0295)
High-income	0.1188^***^	0.1209^***^	0.1202^***^	0.1082^***^	0.1107^***^	0.1100^***^
	(0.0338)	(0.0335)	(0.0338)	(0.0351)	(0.0347)	(0.0350)
Clean Government	−0.0804^***^	−0.0804^***^	−0.0806^***^	−0.0798^***^	−0.0798^***^	−0.0802^***^
	(0.0051)	(0.0053)	(0.0051)	(0.0056)	(0.0058)	(0.0056)
Life chances	0.0958^***^	0.0959^***^	0.0962^***^	0.0990^***^	0.0988^***^	0.0991^***^
	(0.0154)	(0.0147)	(0.0154)	(0.0171)	(0.0163)	(0.0170)
Social values	0.0665^***^	0.0672^***^	0.0669^***^	0.0695^***^	0.0704^***^	0.0699^***^
	(0.0071)	(0.0070)	(0.0070)	(0.0075)	(0.0075)	(0.0074)
Employment	0.0217^**^	0.0220^***^	0.0223^**^	0.0255^***^	0.0261^***^	0.0266^***^
	(0.0086)	(0.0083)	(0.0088)	(0.0092)	(0.0088)	(0.0093)
*Per capita* GDP		0.0036	0.0032		0.0044	0.0042
		(0.0035)	(0.0036)		(0.0042)	(0.0043)
Living conditions		−0.0018	−0.0020		−0.0025	−0.0027
		(0.0030)	(0.0029)		(0.0032)	(0.0031)
CPI		0.0698	0.0751		0.1100	0.1134
		(0.0870)	(0.0847)		(0.0926)	(0.0901)
Age-fixed effect	Yes	No	Yes	Yes	No	Yes
Pseudo R2	0.0297	0.0280	0.0298	0.0298	0.0282	0.0301
Observations	8,414	8,414	8,414	7,606	7,606	7,606

Standard error of robust clustering at the provincial level in parentheses.

*p < 0.10, **p < 0.05, ***p < 0.01.

The empirical results show that COVID-19 increased trust in government among people who were exposed to SARS in adolescence and decreased trust in government among those who were exposed to SARS in adulthood. Taking column (3) as an example, the coefficients of CNCI, CNCI^*^Agegr_2_ and CNCI^*^Agegr_3_ are −0.0597, 0.0713 and 0.0443, respectively, and the total effect coefficients of CNCI^*^Agegr_2_ and CNCI^*^Agegr_3_ are 0.0116 (0.0713–0.0597) and − 0.0154 (0.0443–0.0597), respectively. The positive effect of Chinese successful fight against SARS is that young adolescents’ trust in government is sustained and strong. Niemi and Sobieszek (1977) thought that young people develop the cognitive ability to deal with political thoughts in the later stages of their youth. Trust in government among individuals who were exposed to SARS in adulthood, compared to those who were not exposed to SARS, is less affected by COVID-19. Columns (4)–(6) in Table 3 show the empirical results of excluding the sample data of the provinces that did not experience SARS. The conclusion remains the same.

#### Test of the assumption of DID estimation

The DID in this paper can alleviate endogenous problems to some extent, but it is based on some assumptions. In this section, we need to verify the assumption. Because this paper only involves 1 year’s data, it is impossible to directly test the parallel time trend of DID. Since the fundamental purpose of parallel time trend test is to exclude the influence of other unobservable factors on the estimation, this part used the pseudo treated group and placebo test to indirectly exclude the possible estimation bias caused by other unobservable factors.

#### Pseudo treated group

The first identification hypothesis test adopted is pseudo treated group. The construction of this DID comes from the differences of the severity of COVID-19 epidemic in various provinces and the differences of epidemic experience of different age cohorts. This paper divides the sample into three groups. The first group has not experienced SARS or was under the age of 7 when SARS occurred (i.e., under the age of 24 at the time of the questionnaire survey). This age group is in childhood amnesia stage (“childhood amnesia”), and it is difficult to establish a solid memory before about 7 years old, and cannot form political consciousness. The second group was 7 to 18 years old (i.e., the age range at the time of the questionnaire survey are between 24 and 35). This age group had a stable memory and was forming their values. The government’s successful epidemic prevention eliminated the public’s panic. This event has generated strong memories in the youth, and subconsciously formed their dependence on the government. The last group was 18 years old or older during the SARS period. At this age stage, social values have been basically formed, and their evaluation of events is more calm and objective. According to Chen and Zhou (2007), this paper uses age cohort to construct the difference of an epidemic experience.

In order to test the rationality of the above grouping age cohort, we only retain the samples under 24 years old at the time of the questionnaire, and these sample individuals are basically in childhood amnesia during the SARS epidemic. We divide the retained samples into three groups. The first group is the underage group (i.e., under 18 years old at the time of the questionnaire). The remaining individuals are equally divided into two groups, the age range is [10,18), [18,21), and [21,23) respectively. We use these three pseudo treated groups to replace the three age groups (Agegr) in (1). The regression results are shown in Table 4. It is found that the estimation result of cross multiplication terms are not statistically significant. We also try to group the retained samples into [10,18), [18,20), [20,23) and [10,18), [18,22), [22,23). The regression results of the cross multiplication term still have no statistical significance, which verifies the rationality of the age grouping of this paper.

**Table 4:** Pseudo treated group.

	(1)	(2)	(3)	(4)	(5)	(6)
	TIG	TIG	TIG	TIG	TIG	TIG
CNCI	−0.0483	−0.0359	−0.0456	−0.0357	−0.0231	−0.0360
	(0.0364)	(0.0363)	(0.0367)	(0.0369)	(0.0378)	(0.0375)
CNCI^*^Agegr_2_	0.0232	0.0105	0.0238	0.0103	−0.0034	0.0113
	(0.0378)	(0.0369)	(0.0375)	(0.0355)	(0.0374)	(0.0353)
CNCI^*^Agegr_3_	−0.0197	−0.0320	−0.0209	−0.0275	−0.0433	−0.0279
	(0.0425)	(0.0417)	(0.0424)	(0.0420)	(0.0424)	(0.0421)
Agegr_2_	−0.0133	−0.0845	−0.0065	0.0243	−0.0442	0.0309
	(0.1413)	(0.1292)	(0.1431)	(0.1680)	(0.1388)	(0.1686)
Agegr_3_	0.2337	0.1128	0.2345	0.3565^*^	0.1700	0.3534^*^
	(0.1991)	(0.1210)	(0.1965)	(0.2166)	(0.1290)	(0.2139)
DV	Yes	Yes	Yes	Yes	Yes	Yes
PV	No	Yes	Yes	No	Yes	Yes
AFE	Yes	No	Yes	Yes	No	Yes
Pseudo R^2^	0.0277	0.0272	0.0281	0.0290	0.0285	0.0295
Observations	1,008	1,008	1,008	882	882	882

Standard error of robust clustering at the provincial level in parentheses. Demographic variables are abbreviated as DV. Provincial variables are abbreviated as PV. The age-fixed effect is abbreviated as AFE. The control variables are the same as those in Table 3.

*p < 0.10.

#### Placebo test

Another hypothesis test in this paper is to exclude the influence of other unobservable provincial characteristics that change with age groups. Although the control variables of provinces are also included in the regression, these characteristics may have different effects on people of different age groups, thus affecting the identification hypothesis, which is often beyond the control of existing models. An indirect test (placebo test) is used. We randomly assign the cumulative number of COVID-19 confirmed infections in each province, obtain false data of the cumulative number of COVID-19 confirmed infections, and carry out regressions. The empirical results are shown in Table 5, and the coefficients of cross multiplication term are still not statistically significant. In order to eliminate the accidental results caused by random allocation, this paper repeats this random allocation 500 times and obtains 500 estimation coefficients ${\hat{\beta }}_{2}^{random}$ and ${\hat{\beta }}_{3}^{random}$ respectively. The values of ${\hat{\beta }}_{2}^{random}$and ${\hat{\beta }}_{3}^{random}$ are distributed near zero and are similar to normal distribution. By comparing with the “real” estimation coefficient (the coefficient of regression in column (3) of Table 3), the t-test results show that these two false estimates are significantly different from the “real” estimation coefficient at the 1% statistical level (t-values are −8.6093 and − 6.8685 respectively), which indicates that the estimation equations have passed the placebo tests.

**Table 5:** Placebo test.

	(1)	(2)	(3)	(4)	(5)	(6)
	TIG	TIG	TIG	TIG	TIG	TIG
CNCI	−0.0015	−0.0082	−0.0097	0.0002	−0.0093	−0.0113
	(0.0061)	(0.0180)	(0.0172)	(0.0069)	(0.0173)	(0.0168)
CNCI^*^Agegr_2_	−0.0006	−0.0025	−0.0004	−0.0021	−0.0042	−0.0021
	(0.0059)	(0.0057)	(0.0059)	(0.0066)	(0.0062)	(0.0064)
CNCI^*^Agegr_3_	0.0040	0.0012	0.0042	0.0029	0.0001	0.0029
	(0.0064)	(0.0058)	(0.0063)	(0.0073)	(0.0067)	(0.0071)
Agegr_2_	0.0477	−0.0018	0.0468	0.1612	0.0287	0.1626
	(0.1268)	(0.0375)	(0.1272)	(0.1271)	(0.0399)	(0.1281)
Agegr_3_	−0.3275^***^	0.0436	−0.3640^***^	−0.2206^*^	0.0797^*^	−0.2682^**^
	(0.1209)	(0.0430)	(0.1298)	(0.1222)	(0.0458)	(0.1320)
DV	Yes	Yes	Yes	Yes	Yes	Yes
PV	No	Yes	Yes	No	Yes	Yes
AFE	Yes	No	Yes	Yes	No	Yes
Pseudo R2	0.0296	0.0280	0.0297	0.0298	0.0281	0.0300
Observations	8,414	8,414	8,414	7,606	7,606	7,606

Standard error of robust clustering at the provincial level in parentheses. DV, PV and AFE are the same as those Table 4.

*p < 0.10, **p < 0.05, ***p < 0.01.

### Endogeneity

Although the empirical scheme of DID in this paper has alleviated the endogeneity issue caused by missing variables to a certain extent, there are still endogenous problems caused by two-way causality in logic. The prevention and control of epidemic spread is testing the credibility of the government. The higher the credibility of the government, the more residents may actively implement the government’s epidemic prevention and control measures (Trent et al., 2022), thereby blocking the spread of the epidemic and reducing the severity of the epidemic. That is, CNCI is an endogenous variable. In order to overcome the endogenous impact of two-way causality on the estimation results, we selected “the total number of flights from Wuhan to each province in China every day” as the instrumental variable (IV). This variable was manually compiled from the application program of China’s leading tourism company “Tuniu.” First, we counted the number of daily flights from Wuhan to cities in China, and then summarized the number of flights to each province according to the province of each city.

The number of flights between cities is one of the indicators reflecting the degree of close commercial ties. Wuhan city in Hebei Province was the first city to report the outbreak of COVID-19. Before the closure of Wuhan, according to the mayor of Wuhan at the press conference on epidemic prevention and control, about 5 million people left Wuhan due to the impact of the Spring Festival and the epidemic. COVID-19 has spread rapidly throughout China, with the vast majority of imported cases. The more flights between each province and Wuhan, the more imported cases in the province will be, and the more cases will be infected. Therefore, the IV selected in this paper meets the requirements of correlation. The more flights between provinces, generally reflects the close business contacts between the two places. However, the convenience of transportation does not directly affect residents’ trust in the government, and the IV meets the exogenous requirements.

In order to overcome the endogeneity problem of CNCI, CNCI ^*^ Agegr_2_, and CNCI ^*^ Agegr_3_, and estimate the impact of these variables on trust in government consistently and effectively, this paper uses the conditional mixed process method (CMP) for simultaneous likelihood estimation (Roodman, 2011). Specifically, the simultaneous recursive system consists of equations (1)–(4).


(2) $$\begin{array}{l} CNCI_{p}=\tilde{\alpha }+{\tilde{\beta }}_{1}IV+\overset{3}{\underset{a=2}{\sum }}{\tilde{\beta }}_{a}IV*Agegr_{ia}+\overset{3}{\underset{a=2}{\sum }}{\tilde{\gamma }}_{a}Agegr_{ia}\\ +\lambda_{a}+X_{iap}\tilde{\varphi }+Z_{ip}\tilde{\psi }+u_{1}={\tilde{\theta }}_{ip}+u_{1} \end{array}$$

(3) $$\begin{array}{l} CNCI_{p}*Agegr_{i2}=\overset{⌢}{\alpha }+{\overset{⌢}{\beta }}_{1}IV+\overset{3}{\underset{a=2}{\sum }}{\overset{⌢}{\beta }}_{a}IV*Agegr_{ia}\\ +\overset{3}{\underset{a=2}{\sum }}{\overset{⌢}{\gamma }}_{a}Agegr_{ia}+\lambda_{a}+X_{iap}\overset{⌢}{\varphi }\\ +Z_{ip}\overset{⌢}{\psi }+u_{2}={\overset{⌢}{\theta }}_{ip}+u_{2} \end{array}$$

(4) $$\begin{array}{l} CNCI_{p}*Agegr_{i3}=\overset{⌣}{\alpha }+{\overset{⌣}{\beta }}_{1}IV+\overset{3}{\underset{a=2}{\sum }}{\overset{⌣}{\beta }}_{a}IV*Agegr_{ia}\\ +\overset{3}{\underset{a=2}{\sum }}{\overset{⌣}{\gamma }}_{a}Agegr_{ia}+\lambda_{a}+X_{iap}\overset{⌣}{\varphi }\\ +Z_{ip}\overset{⌣}{\psi }+u_{3}={\overset{⌣}{\theta }}_{ip}+u_{3} \end{array}$$

Assume that the error term *U =* (*u*_1_*, u*_2_*, u*_3_, *ε_iap_*)*^T^* follows the multivariate normal distribution, and *T* stands for vector transposition. That is, the conditional expectation of *U* is equal to zero, and *U* ~ *N*(0, Σ), where


$$\sum =\left[\begin{array}{cccc} 1 & \rho_{12} & \rho_{13} & \rho_{14}\\ \rho_{12} & 1 & \rho_{23} & \rho_{24}\\ \rho_{13} & \rho_{23} & 1 & \rho_{34}\\ \rho_{14} & \rho_{24} & \rho_{34} & 1 \end{array}\right]$$

In the simultaneous recursive system (1)–(4), the estimation coefficients *β*_2_, *β*_3_, ${\tilde{\beta }}_{1}$, ${\hat{\beta }}_{2}$ and ${\overset{⌣}{\beta }}_{3}$ must be statistically significant. The variable *CNCI_p_* is endogenous if *ρ*_14_ is significantly non-zero. Because *CNCI_p_*, CNCI_p_^*^Agegr_2a_ and CNCI_p_^*^Agegr_3*a*_ do not appear in each other’s equations, if the correlation coefficients *ρ*_12_, *ρ*_13_ and *ρ*_23_ between the corresponding error terms are significantly non-zero, the effectiveness of the system estimation will be improved.

The likelihood is


$$\begin{array}{l} \begin{array}{l} L=\left[\Pi_{i}\phi_{1}\left(CNCI_{p}-{\tilde{\theta }}_{ip}\right)\right]\times \left[\Pi_{i}\phi_{2}\left(CNCI_{p}\times Agegr_{2a}-{\overset{⌢}{\theta }}_{ip}\right)\left|\rho_{12}\right.\right]\\ \times \left[\Pi_{i}\phi_{3}(CNCI_{p}\times Agegr_{3a}-{\overset{⌣}{\theta }}_{ip)}\left|\rho_{13},\rho_{23}\right.\right] \end{array}\\ \begin{array}{l} \times \left[\Pi_{j=0}^{10}\left[\Pi_{i,\;Y_{iap}=j}\left(\Phi \right.\left(cut_{j+1}-\theta_{ip}\right)\right.\right]\\ \left.\left.\;\;\;-\Phi \left(cut_{j}-\theta_{ip}\right)\right)\left|\rho_{14},\rho_{24},\rho_{34}\right.\right] \end{array} \end{array}$$

where Π is the multiplication symbol, and Φ is the zero-centered cumulative normal distribution of *ε_iap_*, and *ϕ*_1_, *ϕ*_2_ and *ϕ*_3_ are the zero-centered normal distribution of *u*_1_*, u*_2_ and *u*_3_, respectively.

The calculation of logarithmic form of the maximum likelihood function *L* needs high-dimensional integral arguments, which is difficult to be calculated by common methods. To solve this problem, Roodman (2011) provided the Stata module CMP based on Geweke, Hajivassiliu and Keane (GHK) algorithms. The above recursive equation system guarantees the proper treatment of the endogeneity of CNCI and trust in government.

Table 6 shows the empirical estimation results. The simultaneous estimation results in columns (1)–(4) do not consider the age-fixed effect and macro control variables. Among them, columns (1)–(3) are the regression results of endogenous variables CNCI (resp. CNCI ^*^ Agegr_2_ and CNCI ^*^ Agegr_3_) on its instrumental variable IV (resp. IV ^*^ Agegr_2_ and IV ^*^ Agegr_3_) and other control variables. The estimated coefficients of IV, IV ^*^ Agegr_2_ and IV ^*^ Agegr_3_ are statistically significant at 1% in columns (1), (2) and (3), respectively. The auxiliary estimation parameter atanhrho_14 in columns (1)–(4) is significantly different from 0 at the statistical level of 5%, which indicates that CNCI is indeed an endogenous variable. The estimated results of column (4) in CMP are consistent with the main regression results in Table 3, and the regression results are still robust. Parameter atanhrho_12, atanhrho_13 and atanhrho_23 are significantly different from 0 at the statistical level of 5%, which indicates that the estimation result of CMP simultaneous likelihood method is more consistent and effective than that of single equation estimation. In the simultaneous estimation results of columns (5)–(8), age-fixed effect and macro control variables are added, so we consider age-fixed effect and the situation without macro control variables, and the regression results are still robust.

**Table 6:** Endogeneity test by conditional mixed process method (CMP).

	(1)	(2)	(3)	(4)	(5)	(6)	(7)	(8)
	CMP				CMP			
	CNCI	CNCI^*^Agegr_2_	CNCI^*^Agegr_3_	TIG	CNCI	CNCI^*^Agegr_2_	CNCI^*^Agegr_3_	TIG
	OLS	OLS	OLS	Oprobit	OLS	OLS	OLS	Oprobit
IV	0.0017^***^	−0.0000	0.0000		0.0009^**^	−0.0004	−0.0003	
	(0.0005)	(0.0000)	(0.0000)		(0.0004)	(0.0002)	(0.0002)	
IV^*^Agegr_2_	−0.0001	0.0017^***^	−0.0000		0.0000	0.0017^***^	0.0000	
	(0.0001)	(0.0005)	(0.0000)		(0.0001)	(0.0005)	(0.0000)	
IV^*^Agegr_3_	−0.0002^***^	0.0000	0.0015^***^		−0.0001	0.0001	0.0016^***^	
	(0.0001)	(0.0000)	(0.0005)		(0.0001)	(0.0000)	(0.0005)	
CNCI				−0.0551^***^				−0.0591^***^
				(0.0048)				(0.0065)
CNCI^*^Agegr_2_				0.0690^***^				0.0696^***^
				(0.0053)				(0.0062)
CNCI^*^Agegr_3_				0.0473^***^				0.0422^***^
				(0.0063)				(0.0058)
Agegr_2_	0.0011	0.0262^**^	−0.0016	−0.0158	−0.0061	0.0224^*^	−0.0061^***^	0.0394
	(0.0037)	(0.0124)	(0.0010)	(0.0330)	(0.0040)	(0.0122)	(0.0023)	(0.1262)
Agegr_3_	0.0037	−0.0016	0.0287^**^	0.0359	0.0050	−0.0275^***^	0.0609^***^	−0.3488^***^
	(0.0032)	(0.0016)	(0.0121)	(0.0404)	(0.0091)	(0.0071)	(0.0054)	(0.1220)
Marriage	0.0013	0.0009	0.0003	−0.1613^***^	0.0025	0.0013	0.0009	−0.1469^***^
	(0.0025)	(0.0022)	(0.0006)	(0.0339)	(0.0019)	(0.0018)	(0.0011)	(0.0385)
Health	−0.0007	−0.0006	−0.0000	0.0464^***^	−0.0001	−0.0003	0.0001	0.0460^***^
	(0.0008)	(0.0005)	(0.0004)	(0.0080)	(0.0004)	(0.0004)	(0.0002)	(0.0079)
Happiness	0.0004	−0.0001	0.0005^**^	0.0984^***^	0.0000	−0.0003	0.0004^*^	0.0985^***^
	(0.0005)	(0.0003)	(0.0002)	(0.0056)	(0.0004)	(0.0002)	(0.0002)	(0.0057)
Education	0.0005	0.0001	0.0004	−0.0007	0.0003	0.0000	0.0003^*^	−0.0004
	(0.0003)	(0.0001)	(0.0002)	(0.0078)	(0.0002)	(0.0001)	(0.0002)	(0.0082)
Gender	−0.0021^***^	−0.0004	−0.0014^***^	0.0060	−0.0003	0.0003	−0.0007^**^	0.0076
	(0.0008)	(0.0006)	(0.0004)	(0.0181)	(0.0004)	(0.0005)	(0.0003)	(0.0190)
Medical Insurance	−0.0078^**^	−0.0044^***^	−0.0018	0.1091^***^	0.0002	−0.0004	0.0013	0.1151^***^
	(0.0033)	(0.0017)	(0.0012)	(0.0320)	(0.0018)	(0.0009)	(0.0009)	(0.0318)
Working Income	0.0015^***^	0.0008^***^	0.0006^***^	0.0055^*^	0.0002	0.0002^**^	0.0001	0.0050
	(0.0003)	(0.0002)	(0.0001)	(0.0029)	(0.0001)	(0.0001)	(0.0001)	(0.0032)
low-income	0.0018	−0.0001	0.0013^**^	−0.1025^***^	−0.0004	−0.0010	0.0004	−0.1066^***^
	(0.0011)	(0.0008)	(0.0007)	(0.0310)	(0.0006)	(0.0008)	(0.0006)	(0.0316)
High-income	−0.0033^**^	−0.0013	−0.0022^***^	0.1195^***^	−0.0011	−0.0003	−0.0013^*^	0.1202^***^
	(0.0013)	(0.0010)	(0.0007)	(0.0336)	(0.0009)	(0.0009)	(0.0007)	(0.0337)
Clean Government	0.0005	0.0003^*^	0.0002	−0.0801^***^	−0.0001	0.0000	−0.0000	−0.0806^***^
	(0.0003)	(0.0002)	(0.0001)	(0.0052)	(0.0002)	(0.0001)	(0.0001)	(0.0051)
Life Chances	0.0008	0.0010^*^	−0.0001	0.0954^***^	0.0002	0.0008^**^	−0.0004	0.0962^***^
	(0.0010)	(0.0006)	(0.0006)	(0.0147)	(0.0005)	(0.0004)	(0.0004)	(0.0153)
Social Values	−0.0003	−0.0000	−0.0002	0.0668^***^	−0.0001	0.0001	−0.0002	0.0669^***^
	(0.0004)	(0.0003)	(0.0002)	(0.0071)	(0.0002)	(0.0002)	(0.0001)	(0.0070)
Employment	−0.0014^***^	−0.0009^***^	−0.0005^**^	0.0214^***^	−0.0003	−0.0003^***^	−0.0001	0.0223^**^
	(0.0004)	(0.0002)	(0.0002)	(0.0081)	(0.0002)	(0.0001)	(0.0001)	(0.0088)
PerCapitaGDP					0.0026^*^	0.0014^**^	0.0010	0.0032
					(0.0014)	(0.0007)	(0.0006)	(0.0032)
Living Conditions					0.0026^**^	0.0012^**^	0.0010^**^	−0.0020
					(0.0010)	(0.0005)	(0.0004)	(0.0020)
CPI					0.0181	0.0116	0.0050	0.0755
					(0.0192)	(0.0082)	(0.0083)	(0.0548)
Constant	0.0245^*^	0.0036	−0.0040		−1.8636	−1.1953	−0.5209	
	(0.0125)	(0.0039)	(0.0034)		(1.9662)	(0.8391)	(0.8523)	
atanhrho_12	0.8476^***^ (0.0323)				0.5979^***^ (0.0816)			
atanhrho_13	0.7160^***^ (0.0213)				0.5177^***^ (0.0728)			
atanhrho_14	0.0265^**^ (0.0107)				0.0220^*^ (0.0122)			
atanhrho_23	−0.0238^**^ (0.0117)				−0.3387^***^ (0.1116)			
atanhrho_24	0.0177 (0.0116)				0.0117 (0.0136)			
atanhrho_34	0.0215^**^ (0.0103)				0.0158^*^ (0.0092)			
Observations	8,414				8,414			

Standard error of robust clustering at the provincial level in parentheses.

*p < 0.10, **p < 0.05, ***p < 0.01.

### Heterogeneity analysis

This part studies the heterogeneous impact of COVID-19 on residents’ trust in government from the perspectives of social position, relative income, sex, work and regions. Among them, social position is obtained from responses to the question “How do you rate your social status in the local area?” Answers to the questionnaire are divided into five levels, with 1 indicating very low and 5 indicating very high. When the score is 1–3, the social position = 1; when the score is 4–5, the social position = 0. Relative income is obtained according to the question “How do you rate your income in the local area?” The answer has five levels, where 1 indicates very low and 5 indicates very high. When the score is 1–3, relative income = 1; when the score is 4–5, relative income = 0.

We study the heterogeneity of the impact of COVID-19 on individuals’ trust in government by constructing a triple difference. The regression results are shown in Table 7. The regression results based on heterogeneous social position are reported in column (1) of Table 7. The CINI^*^dummy coefficient is significantly negative, indicating that for people with low social status who were not exposed to SARS, the impact of COVID-19 on their trust in government is negative. The coefficient of CNCI^*^Agegr_2_^*^dummy is positive, which indicates that the COVID-19 epidemic has a positive impact on the trust of the low social status groups who were exposed to SARS in adolescence. The coefficient of CNCI^*^Agegr_3_^*^dummy is significantly positive, but the total effect is negative, indicating that the COVID-19 epidemic has a negative impact on the trust of the low social status groups who were exposed to SARS in adulthood, and the negative effect is much smaller. Column (2) reports the empirical results based on the heterogeneity of subjective relative income. The results show that the COVID-19 epidemic has a negative effect on the trust of the relatively low-income groups that did not experience SARS but a positive effect on the trust of the low-income groups that were exposed to SARS in adolescence and a small negative effect on the low-income group exposed to SARS in adulthood. Column (3) reports the regression results for sex classification. Compared with women, COVID-19 has a significant negative effect on trust in government of men who were not exposed to SARS and a positive effect on trust in government of men who were exposed to SARS in adolescence, and a small negative effect on trust in government of men who were exposed to SARS in adulthood. The COVID-19 epidemic increases people’s unemployment risk. Friehe and Marcus (2021) found that involuntary unemployment reduced trust by approximately 9% of the standard deviation. Column (4) shows that the COVID-19 epidemic has reduced the trust of people with jobs and no experience of SARS, increased the trust of people with jobs and experienced SARS in adolescence, and reduced the trust of people with jobs and experienced SARS in adulthood. Compared with urban residents, rural residents trust grass-roots institutions more (Lo et al., 2016), while rural migrants and urban migrants have lower trust in local governments (Niu and Zhao, 2018). Column (5) reports empirical results based on regional heterogeneity. Compared to rural areas, the COVID-19 epidemic has increased the trust of urban residents who were exposed to SARS in adolescence. However, for urban residents who were not exposed to SARS, the impact of the COVID-19 epidemic has decreased their trust in government.

**Table 7:** Heterogeneity analysis.

	(1)	(2)	(3)	(4)	(5)
	Social position(dummy = 1: low)	Relative income(dummy = 1: low)	Sex(dummy = 1: male)	Work(dummy = 1: yes)	Regions(dummy = 1: urban)
CNCI^*^dummy	−0.0947^***^	−0.0648^***^	−0.0450^***^	−0.0923^***^	−0.0722^***^
	(0.0087)	(0.0072)	(0.0087)	(0.0107)	(0.0146)
CNCI^*^Agegr_2_^*^dummy	0.1007^***^	0.0848^***^	0.0505^***^	0.0969^***^	0.0835^***^
	(0.0071)	(0.0075)	(0.0087)	(0.0112)	(0.0140)
CNCI^*^Agegr_3_^*^dummy	0.0707^***^	0.0463^***^	0.0391^***^	0.0768^***^	0.0739^***^
	(0.0066)	(0.0060)	(0.0093)	(0.0080)	(0.0132)
Agegr_2_	0.0387	0.0391	0.0446	0.2501	0.0222
	(0.1277)	(0.1279)	(0.1280)	(0.2484)	(0.1188)
Agegr_3_	−0.3510^***^	−0.3483^***^	−0.3468^***^	−0.1400	−0.3582^***^
	(0.1221)	(0.1223)	(0.1224)	(0.2510)	(0.1195)
DV	Yes	Yes	Yes	Yes	Yes
PV	Yes	Yes	Yes	Yes	Yes
AFE	Yes	Yes	Yes	Yes	Yes
Pseudo R^2^	0.0298	0.0298	0.0297	0.0295	0.0292
Observations	8,411	8,414	8,414	8,105	8,087

Standard error of robust clustering at the provincial level in parentheses. DV, PV and AFE are the same as those in Table 5.

***p < 0.01.

## Robustness

### Changing explanatory variables

We use the provincial infection rate (IR) and provincial death toll (DT) of COVID-19 to replace the CNCI for the robustness test. The infection rate is calculated by dividing the CNCI on July 1, 2020 by the total population in each province (IR is equal to the CNCI divided by the provincial population in millions). Columns (1)–(4) in Table 8 are the regression results using IR as the explained variable, and columns (5)–(8) are the regression results using the provincial death toll (Divide by 100, abbreviated as DT) as the explained variable. The population size of the province is considered, so IR is used as an explanatory variable. Because the virus is transmitting in society, provinces with larger population generally have a higher infection rate, so it is reasonable to use IR. Similarly, the number of deaths due to COVID-19 is also related to the population of the province, and the number of deaths in the province also reflects the severity of the epidemic. Therefore, DT is used as an explanatory variable for robustness testing.

**Table 8:** Robustness test: Changing explanatory variables.

	(1)	(2)	(3)	(4)		(5)	(6)	(7)	(8)
	TIG	TIG	TIG	TIG		TIG	TIG	TIG	TIG
IR	−3.4271^***^	−3.6904^***^	−3.3038^***^	−3.6379^***^	DT	−0.0081^***^	−0.0088^***^	−0.0079^***^	−0.0087^***^
	(0.3923)	(0.5408)	(0.3644)	(0.5370)		(0.0007)	(0.0011)	(0.0007)	(0.0011)
IR^*^Agegr_2_	4.3994^***^	4.4139^***^	4.2981^***^	4.2967^***^	DT^*^Agegr_2_	1.0277^***^	1.0313^***^	1.0000^***^	0.9993^***^
	(0.5262)	(0.5282)	(0.5171)	(0.5149)		(0.0902)	(0.0907)	(0.0856)	(0.0850)
IR^*^Agegr_3_	2.7088^***^	2.7221^***^	2.6379^***^	2.6386^***^	DT^*^Agegr_3_	0.6341^***^	0.6372^***^	0.6180^***^	0.6180^***^
	(0.4632)	(0.4676)	(0.4798)	(0.4770)		(0.0775)	(0.0776)	(0.0854)	(0.0833)
Agegr_2_	0.0397	0.0395	0.1516	0.1536	Agegr_2_	0.0438	0.0437	0.1556	0.1576
	(0.1274)	(0.1283)	(0.1278)	(0.1293)		(0.1276)	(0.1284)	(0.1279)	(0.1295)
Agegr_3_	−0.3299^***^	−0.3488^***^	−0.2249^*^	−0.2483^**^	Agegr_3_	−0.3273^***^	−0.3462^***^	−0.2224^*^	−0.2458^**^
	(0.1201)	(0.1226)	(0.1200)	(0.1226)		(0.1200)	(0.1225)	(0.1200)	(0.1225)
DV	Yes	Yes	Yes	Yes	DV	Yes	Yes	Yes	Yes
PV	No	Yes	No	Yes	PV	No	Yes	No	Yes
AFE	Yes	Yes	Yes	Yes	AFE	Yes	Yes	Yes	Yes
Pseudo R^2^	0.0297	0.0298	0.0298	0.0301	Pseudo R^2^	0.0297	0.0298	0.0298	0.0301
Observations	8,414	8,414	7,606	7,606	Observations	8,414	8,414	7,606	7,606

Standard error of robust clustering at the provincial level in parentheses. DV, PV and AFE are the same as those in Table 5.

*p < 0.10, **p < 0.05, ***p < 0.01.

The results show that the impact of COVID-19 on individuals’ trust in government is robust. The regression samples in columns (3)–(4) of Table 8 exclude provinces without SARS, and the regression samples in columns (7)–(8) also remove provinces without SARS. The results are all robust.

### Changing the empirical model

This part uses ordered probit, ordered logit and tobit models for the robustness test, and the regression results are still robust (see Table 9).

**Table 9:** Robustness test: Using other empirical models.

	Ologit			Tobit		
	(1)	(2)	(3)	(4)	(5)	(6)
CINI	−0.1045^***^	−0.1063^***^	−0.1030^***^	−0.1260^***^	−0.1257^***^	−0.1240^***^
	(0.0125)	(0.0128)	(0.0138)	(0.0180)	(0.0196)	(0.0197)
CNCI^*^Agegr_2_	0.1304^***^	0.1329^***^	0.1268^***^	0.1559^***^	0.1559^***^	0.1504^***^
	(0.0102)	(0.0106)	(0.0104)	(0.0148)	(0.0179)	(0.0166)
CNCI^*^Agegr_3_	0.0881^***^	0.0807^***^	0.0748^***^	0.1003^***^	0.0877^***^	0.0844^***^
	(0.0096)	(0.0114)	(0.0111)	(0.0154)	(0.0166)	(0.0172)
Agegr_2_	−0.0085	0.1301	0.3105	−0.0416	0.0656	0.3507
	(0.0542)	(0.2198)	(0.2280)	(0.0770)	(0.3147)	(0.3090)
Agegr_3_	0.0807	−0.4440^**^	−0.2664	0.0719	−0.7174^**^	−0.4698
	(0.0630)	(0.2074)	(0.2056)	(0.0936)	(0.3025)	(0.3007)
DV	Yes	Yes	Yes	Yes	Yes	Yes
PV	Yes	Yes	Yes	Yes	Yes	Yes
AFE	No	Yes	Yes	No	Yes	Yes
Constant				−15.7855	−16.8023	−26.4680
				(20.7510)	(20.1610)	(21.1939)
Pseudo R^2^	0.0299	0.0318	0.0320	0.0253	0.0270	0.0273
Observations	8,414	8,414	7,606	8,414	8,414	7,606

Standard error of robust clustering at the provincial level in parentheses. DV, PV and AFE are the same as those in Table 5.

**p < 0.05, ***p < 0.01.

### Adding control variables

In this part, we add control variables that may affect trust in government for the robustness test. The control variables mainly include some subjective problems. Specific control variables and settings are as follows: rich-poor gap variable, which is obtained from the question “How serious do you think the gap between the rich and the poor is in China?” (0 means very not serious, and 10 means very serious). The social security variable is obtained from the question “How serious do you think the social security problem is in China?” (0 means very not serious, and 10 means very serious). We add the above variables in columns (1)–(6) of Table 10 in turn. The robustness of the empirical results is verified again.

**Table 10:** Robustness test: Add control variables.

	(1)	(2)	(3)	(4)	(5)	(6)
	TIG	TIG	TIG	TIG	TIG	TIG
CNCI	−0.0546^***^	−0.0594^***^	−0.0596^***^	−0.0542^***^	−0.0590^***^	−0.0591^***^
	(0.0048)	(0.0074)	(0.0081)	(0.0048)	(0.0074)	(0.0081)
CNCI^*^Agegr_2_	0.0702^***^	0.0703^***^	0.0711^***^	0.0696^***^	0.0697^***^	0.0703^***^
	(0.0061)	(0.0061)	(0.0073)	(0.0062)	(0.0062)	(0.0075)
CNCI^*^Agegr_3_	0.0487^***^	0.0491^***^	0.0438^***^	0.0484^***^	0.0488^***^	0.0436^***^
	(0.0062)	(0.0062)	(0.0067)	(0.0062)	(0.0062)	(0.0067)
Agegr_2_	−0.0140	−0.0126	0.0425	−0.0142	−0.0128	0.0439
	(0.0334)	(0.0334)	(0.1281)	(0.0332)	(0.0333)	(0.1294)
Agegr_3_	0.0371	0.0372	−0.3514^***^	0.0370	0.0372	−0.3343^**^
	(0.0404)	(0.0398)	(0.1229)	(0.0403)	(0.0397)	(0.1303)
Rich-poor gap	−0.0047	−0.0052	−0.0049	−0.0043	−0.0048	−0.0046
	(0.0095)	(0.0095)	(0.0089)	(0.0096)	(0.0095)	(0.0089)
Social security				−0.0049	−0.0050	−0.0051
				(0.0072)	(0.0072)	(0.0070)
DV	Yes	Yes	Yes	Yes	Yes	Yes
PV	No	Yes	Yes	No	Yes	Yes
AFE	No	No	Yes	No	No	Yes
Pseudo R2	0.0279	0.0280	0.0298	0.0279	0.0280	0.0298
Observations	8,408	8,408	8,408	8,401	8,401	8,401

Standard error of robust clustering at the provincial level in parentheses. DV, PV and AFE are the same as those in Table 5.

**p < 0.05, ***p < 0.01.

### Changing age range

Considering the different division of the growth stages before adulthood. In this part, we change the age range of childhood and adolescence to a small extent. First, the medical profession generally believes that children’s puberty begins at the age of 10, so we define the age before 10 as childhood, the age between 10 and 18 as adolescence, and the age above 18 as adulthood. The empirical results are shown in columns 1–3 of Table 11. Second, we shorten the age range of childhood, and defined it as under 5 years old, puberty from 5 to 18 years old, and adulthood above 18 years old. The empirical results are shown in columns 4–6 of Table 11. Same as above, we classify those who were not born during the SARS period and those who were in childhood as the same group, which is also the reference group for this paper.

**Table 11:** Robustness test: Changing the age range.

	(1)	(2)	(3)	(4)	(5)	(6)
	TIG	TIG	TIG	TIG	TIG	TIG
CNCI	−0.0393^***^	−0.0447^***^	−0.0436^***^	−0.0490^***^	−0.0541^***^	−0.0553^***^
	(0.0056)	(0.0073)	(0.0077)	(0.0068)	(0.0080)	(0.0096)
CNCI^*^Agegr_2_	0.0556^***^	0.0564^***^	0.0543^***^	0.0583^***^	0.0588^***^	0.0607^***^
	(0.0046)	(0.0043)	(0.0058)	(0.0088)	(0.0088)	(0.0106)
CNCI^*^Agegr_3_	0.0338^***^	0.0347^***^	0.0281^***^	0.0434^***^	0.0441^***^	0.0399^***^
	(0.0055)	(0.0055)	(0.0055)	(0.0078)	(0.0079)	(0.0096)
Agegr_2_	−0.0611^***^	−0.0621^***^	0.0403	−0.0244	−0.0253	0.0406
	(0.0179)	(0.0179)	(0.1278)	(0.0476)	(0.0477)	(0.1279)
Agegr_3_	0.0009	−0.0009	−0.3486^***^	0.0265	0.0246	−0.3481^***^
	(0.0353)	(0.0353)	(0.1225)	(0.0524)	(0.0525)	(0.1223)
DV	Yes	Yes	Yes	Yes	Yes	Yes
PV	No	Yes	Yes	No	Yes	Yes
AFE	No	No	Yes	No	No	Yes
Constant	10.6034	8.9643	−1.7770	−1.7770	9.8375	8.9457
	(17.0046)	(16.5185)	(18.2406)	(18.2406)	(16.5241)	(16.5026)
Pseudo R2	0.0280	0.0281	0.0297	0.0279	0.0280	0.0297
Observations	8,414	8,414	8,414	8,414	8,414	8,414

Standard error of robust clustering at the provincial level in parentheses. DV, PV and AFE are the same as those in Table 5.

***p < 0.01.

Table 11 shows that the regression results again verify that the COVID-19 epidemic significantly reduced trust in government among people who were not exposed to SARS, but it has even positive impact on trust in government of people who experience SARS in adolescence, and only a weak negative impact on trust in government of people who experience SARS in adulthood.

### Further analysis

This part attempts to explore the mechanism of the impact of major public health emergencies on residents’ trust in government, analyzing the mechanism from two aspects: life chances [see columns (1)–(3) of Table 12] and social values [see columns (4)–(6) of Table 12].

**Table 12:** Mechanism analysis.

	(1)	(2)	(3)	(4)	(5)	(6)
	Oprobit	Oprobit	Oprobit	Oprobit	Oprobit	Oprobit
	Life chances	Life chances	Life chances	Social values	Social values	Social values
CNCI	−0.0560^***^	−0.1226^***^	−0.1107^***^	−0.0355^***^	−0.0240^***^	−0.0273^***^
	(0.0084)	(0.0085)	(0.0150)	(0.0099)	(0.0073)	(0.0086)
CNCI^*^Agegr_2_	0.0499^***^	0.1233^***^	0.1149^***^	0.0350^***^	0.0333^***^	0.0345^***^
	(0.0102)	(0.0107)	(0.0174)	(0.0087)	(0.0058)	(0.0090)
CNCI^*^Agegr_3_	0.0457^***^	0.1231^***^	0.1093^***^	0.0490^***^	0.0442^***^	0.0478^***^
	(0.0087)	(0.0100)	(0.0147)	(0.0101)	(0.0070)	(0.0101)
Agegr_2_	−0.2114^**^	−0.0393	−0.3506^*^	0.2426	0.0149	0.2504
	(0.1074)	(0.0513)	(0.2023)	(0.2400)	(0.0467)	(0.2444)
Agegr_3_	0.3367^***^	−0.2338^***^	0.2441	−0.0509	0.1134^**^	−0.0234
	(0.1177)	(0.0563)	(0.2168)	(0.2143)	(0.0536)	(0.2231)
DV	Yes	Yes	Yes	Yes	Yes	Yes
PV	No	Yes	Yes	No	Yes	Yes
AFE	Yes	No	Yes	Yes	No	Yes
Pseudo R2	0.0892	0.0969	0.0954	0.0562	0.0521	0.0571
Observations	8,414	8,105	8,105	8,105	8,105	8,105

Standard error of robust clustering at the provincial level in parentheses. DV, PV and AFE are the same as those in Table 5.

*p < 0.10, **p < 0.05, ***p < 0.01.

The empirical results show that the COVID-19 epidemic mainly reduces the life chances and social values of people who have not been exposed to SARS and improves the life chances and social values of people who have been exposed to SARS. The literature considers that improving life satisfaction will increase trust in government (Helliwell and Huang, 2008; Weber et al., 2017). COVID-19 may affect people’s trust in government by affecting their life chances and social values.

## Discussion and conclusion

The fear and unease caused by the rapid spread of viruses may envelope the whole society. Rampant epidemics test a country’s prevention and control ability and government credibility. Now, the impact of major public health emergencies on residents’s trust in government is different across countries. Some literature found that the spread of major epidemics will directly lead to a decline in the public’s trust in government (Bangerter et al., 2012; Quinn et al., 2013; Kimhi et al., 2020), More literature showed that trust in government is related to the implementation of governments’ proactive epidemic prevention measures (Groeniger et al., 2021). The existing research mostly focuses on the short-term effect between trust in government and the severity of major infectious diseases. But epidemics have the long-term effect on trust in the government. If past epidemics are a guide, COVID-19 may not have serious impact on trust in government. This may be the reason why COVID-19 has inconsistent impacts on residents’ trust in government.

Although this paper presents some interesting results on the effect of major public health emergencies, this study is not without limitations. CFPS data only publishes the name of the province where the sample individuals are located, but not the real name of the city where they are located. Therefore, this paper studies the impact of epidemics on trust in government from the provincial level. Based on the fact that epidemics prevention and control measures of local governments in China have obvious differences among provinces (for example, each province has its own electronic health pass card, provincial control requirements, etc.), we believe that this will not affect the conclusions of this paper. However, due to the inability to identify the city names in the CFPS data, it is not possible to characterize the differences at the municipal level, and the research conclusions are relatively rough. A detailed study of the impact of major epidemics at the municipal level is an interesting topic for further research.

**Figure 5:**
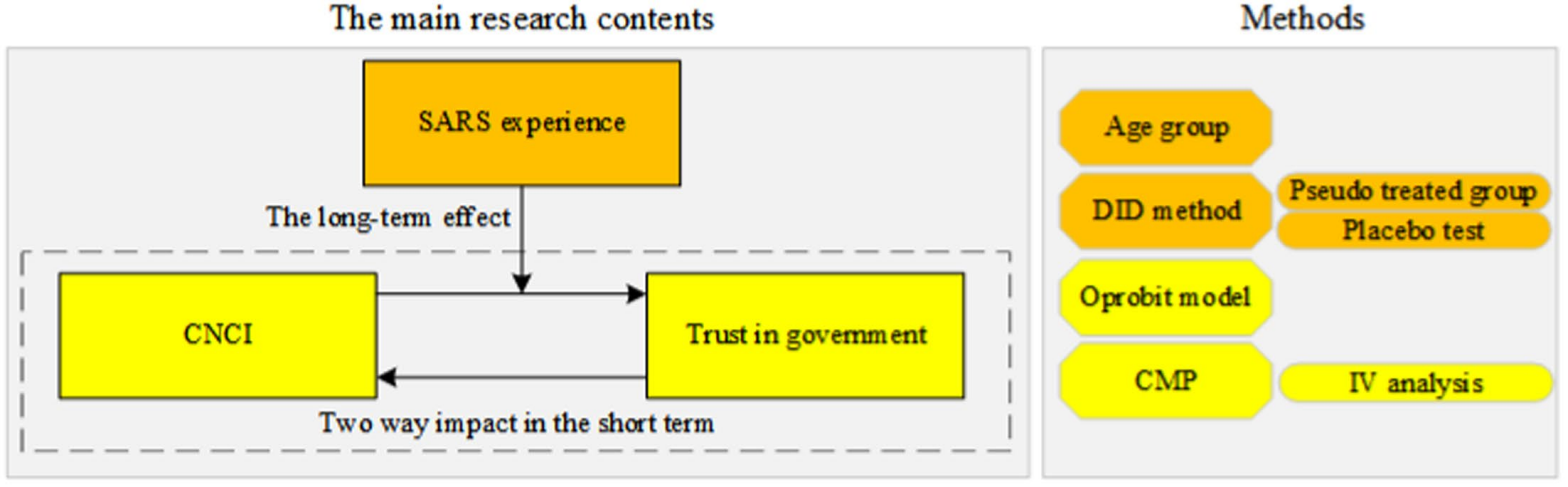
Logical relations and methods.

This research is of great significance. Since 2019, the world has been suffering from the impact of COVID-19. COVID-19 has the potential to reshape all aspects of our society. It not only severely damages the economy and threatens people’s health, but also affects the public’s trust in government. The difference of trust in government may be a good solution to the social dilemma logic behind the spread of coronavirus disease. From the perspective of two major infectious disease outbreaks in China, the impact of major public health emergencies on trust in government is studied. Our research shows that epidemics have the long-term effect on trust in government. It is not possible to accurately judge the impact of one epidemic on trust in government based only on past epidemics. When major infectious disease outbreaks occur again, the public will continue to believe in the government’s ability to curb the virus based on previous successes, which alleviates the impact of COVID-19 on the public’s trust in government. The logical relations between the epidemics and trust in government are shown in Figure 5.

Our research reveals that the COVID-19 epidemic mainly reduced the trust in government of the people who did not experience the epidemic. This indicates that the public trust in government can be shaped by their experiences with government’s success of fighting an epidemic and is lasting. This may also explain why China has been so successful in fighting COVID-19. Interestingly, this paper also attempts to explore the mechanism of the COVID-19 epidemic on the public’s trust in government from two aspects: life chances and social values.

This research provides us with a more comprehensive understanding of the impact of major public health emergencies on the public’s trust in government, pointing out that the trust created by the government’s successful anti-epidemic measures is long-lasting, and the effectiveness of the government’s anti-epidemic measures creates a virtuous cycle. The evidence also proves the importance of decisive measures taken by the government to combat an epidemic from the perspective of trust in government. This study emphasizes the persistence of trust, resilience and vulnerability in the face of adversity and has a positive impact on how countries respond to global public health crises.

## Data availability statement

The data analyzed in this study has been taken from third parties; Real-Time Big Data, Official Data, and the China Family Panel Studies (CFPS). The CFPS data can be found online http://www.isss.pku.edu.cn/cfps/en/data/public/index.htm. Further inquiries regarding this data can be directed to the corresponding author, GZ at gqzhao@sdufe.edu.cn.

## Ethics statement

CFPS follows international guidelines and was conducted by the Peking University of China. The China Social Survey Center informed all respondents of relevant matters before conducting the survey in accordance with the requirements of the Research Ethics Review of China. Participants provided their written informed consent to the survey and permission was given to publish their data in an anonymized manner. For the current study, ethical review and approval, and written informed consent, were not required in accordance with the local legislation and institutional requirements.

## Author contributions

KZ and XY contributed to the conception and design of the study. KZ collected the data and contributed to the analysis of the results and to the writing of the manuscript. GZ performed the statistical analysis. XY supervised the study design and the manuscript draft. All authors contributed to the article and approved the submitted version.

## Funding

This study was supported by the National Natural Science Foundation Project (71871129), the National Social Science Fund Key Project (21AJY007) and Jinan Social Science Fund Project (JNSK22C43).

## Conflict of interest

The authors declare that the research was conducted in the absence of any commercial or financial relationships that could be construed as a potential conflict of interest.

## Publisher’s note

All claims expressed in this article are solely those of the authors and do not necessarily represent those of their affiliated organizations, or those of the publisher, the editors and the reviewers. Any product that may be evaluated in this article, or claim that may be made by its manufacturer, is not guaranteed or endorsed by the publisher.
